# Recognizing Full-Body Exercise Execution Errors Using the Teslasuit

**DOI:** 10.3390/s21248389

**Published:** 2021-12-15

**Authors:** Polona Caserman, Clemens Krug, Stefan Göbel

**Affiliations:** Research Group Serious Games, Technical University of Darmstadt, Rundeturmstrasse 10, 64283 Darmstadt, Germany; clemens.krug@stud.tu-darmstadt.de (C.K.); stefan_peter.goebel@tu-darmstadt.de (S.G.)

**Keywords:** human exercise assessment, full-body motion capture, haptic feedback, imitation learning, transcutaneous electrical nerve stimulation, inertial measurement units

## Abstract

Regular physical exercise is essential for overall health; however, it is also crucial to mitigate the probability of injuries due to incorrect exercise executions. Existing health or fitness applications often neglect accurate full-body motion recognition and focus on a single body part. Furthermore, they often detect only specific errors or provide feedback first after the execution. This lack raises the necessity for the automated detection of full-body execution errors in real-time to assist users in correcting motor skills. To address this challenge, we propose a method for movement assessment using a full-body haptic motion capture suit. We train probabilistic movement models using the data of 10 inertial sensors to detect exercise execution errors. Additionally, we provide haptic feedback, employing transcutaneous electrical nerve stimulation immediately, as soon as an error occurs, to correct the movements. The results based on a dataset collected from 15 subjects show that our approach can detect severe movement execution errors directly during the workout and provide haptic feedback at respective body locations. These results suggest that a haptic full-body motion capture suit, such as the Teslasuit, is promising for movement assessment and can give appropriate haptic feedback to the users so that they can improve their movements.

## 1. Introduction

Sufficient physical activity has significant health benefits, improves overall well-being, and also decreases the general risk of death [1]. Various professional societies recommend guidelines on physical activity and sedentary behavior required to offer significant health benefits and mitigate health risks [2,3]. Although it is generally well known that physical activity is crucial for a healthy lifestyle, more than 80% of the world’s adults and adolescents are still insufficiently active [1,3]. Guthold et al. [4] further observe that physical activity decreased over time in high-income countries. These results show the importance of motivating people to become more physically active. However, to improve health outcomes, it is not only necessary to engage in regular physical activity, but it is also essential to perform sport exercises correctly and prevent injuries due to improper exercise execution.

Especially for movement training during rehabilitation, it is crucial to detect execution errors and help users correct them. According to Statista [5], functional training, as well as rehabilitation and prevention, are among the top trends in personal training in Germany. However, a recent report by the World Health Organization shows that, among others, due to insufficient funding and too few trained rehabilitation professionals, more than 50% of people do not receive the rehabilitation services they require [6]. The COVID-19 pandemic further increased the need for rehabilitation, aggravating the problem even more [6]. This unmet demand increases the need for a system or tool for the automated assessment of execution quality during sports activity, thus supporting or sustaining personal human trainers.

Recent advances and rapid development in sensor technology, such as motion capture systems or wearable devices, instigated interest in the field of human activity recognition [7,8,9,10]. Thereby, both vision- or sensor-based approaches are feasible for various purposes, particularly exergames [11,12,13,14]. Although many previous studies focus on activity recognition to verify whether a user performs a specific exercise, they fail to assess the physical exercises’ quality or performance. Though, quality motion assessment can provide valuable insights and is highly beneficial, especially with physical exercises.

One possibility to assess movements is to leverage the power of machine learning and classify whether a user executes an exercise well or not, as demonstrated in [15,16]. However, such an approach classifies movements merely in discrete classes (e.g., into proper and poor exercise execution) and lacks qualitative feedback on letting users know how to improve a movement. Another possibility is to train machine learning models additionally with improper activity execution, i.e., specifying common movement failures, as proposed by [17,18,19,20]. Although such an approach enables movement correction, it requires that all possible or common execution errors are known a priori. A different approach is to assess individual joint movements in order to provide feedback on the respective joints, as shown in [14,21,22].

Previous works on human movement assessment show promising results; however, they are often limited by evaluating only movements of particular body parts or execution errors need to be known a priori. Another disadvantage of previous works using classification methods is that execution errors can first be recognized after the exercise (partial) repetition. To overcome these limitations and provide immediate feedback during the motion, we propose a system that analyzes exercise execution of a full body, recognizes execution errors throughout the motion, and assists users in correcting those errors in real-time. Thereby, we employ the Teslasuit (Teslasuit: https://teslasuit.io, last accessed on 29 November 2021), a full-body motion capture suit with haptic feedback. We use the sensor data (i.e., accelerometer and gyroscope data as well as derived joint position) of 10 inertial measurements units (IMUs) to recognize and assess full-body movements. The Teslasuit further features haptic feedback by stimulating nerves through the skin with electric impulses to create a range of sensations.

We do not intend to recognize specific exercise execution errors but instead aim to automatically detect arbitrary errors that vary from the intended execution. Apart from giving only auditory or visual feedback and providing feedback first after an exercise execution, we detect execution errors directly and provide haptic feedback already during the execution. Furthermore, in contrast to several previous studies, tracking or monitoring only certain body parts [15,16,18,22,23], we analyze multi-joint movements and thus include the sensor data of legs, arms, and spine. In other words, due to the precise motion capture of individual body parts, we recognize full-body movements in real-time. Moreover, we provide immediate haptic feedback at respective body locations so that users can correct exercise execution errors and improve their movements.

## 2. Materials and Methods

**Figure 1:**
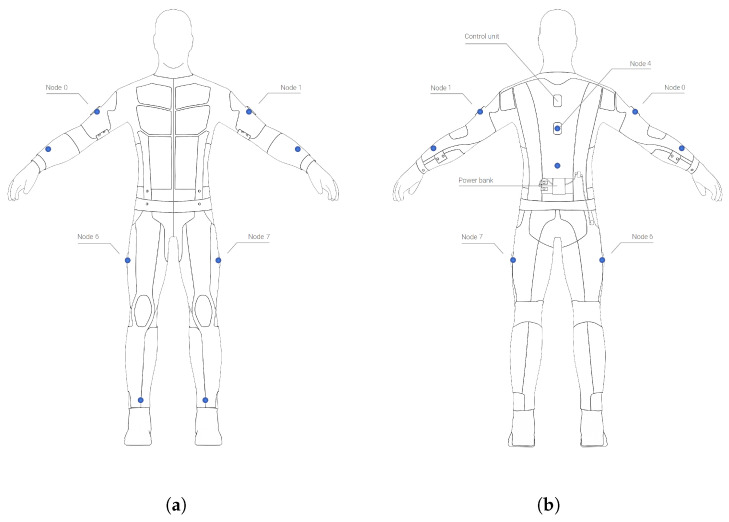
Inertial measurement units (blue dots) located on the Teslasuit (https://teslasuit.io, last accessed on 29 November 2021), (**a**) Front, (**b**) Back.

As discussed in the Introduction, there is currently a gap in the literature regarding movement assessment in real-time and providing full-body haptic feedback. Current approaches are often limited by the potential number of activity execution errors or neglect accurate recognition of individual body parts. Therefore, we aim to develop a full-body exercise recognition and motion assessment system. Towards this end, we employ the Teslasuit and track the legs (thigh and shin), hips, torso, as well as arms (shoulder and forearm) using 10 IMUs, as depicted in Figure 1.

**Figure 2:**
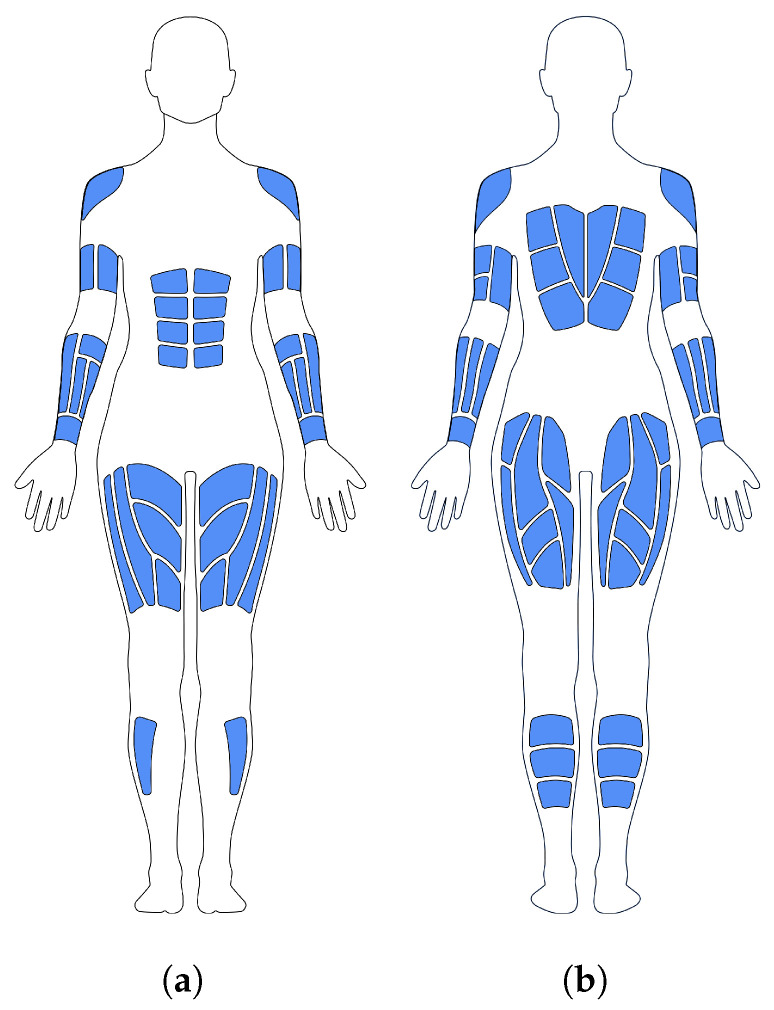
Haptic feedback areas on the Teslasuit (https://teslasuit.io, last accessed on 29 November 2021), (**a**) Front, (**b**) Back.

In addition to motion capture, the system should further provide full-body haptic feedback throughout the motion, similar to the physical touch of a personal trainer. To tackle this challenge, we explore transcutaneous electrical nerve stimulation (TENS) system with 80 electrotactile areas or channels, as detailed in Figure 2, enabling us to provide haptic feedback at relevant body locations. Each electrotactile channel can be controlled individually, simulating different sensations by manipulating pulse width, amplitude, and frequency.

Alternatively, we could use another motion capture system currently available on the market, such as OptiTrack (https://optitrack.com, last accessed on 29 November 2021), Vicon (https://www.vicon.com, last accessed on 29 November 2021), or Perception Neuron (https://neuronmocap.com, last accessed on 29 Novembe 2021) in combination with a haptic system, such as Woojer (https://www.woojer.com, last accessed on 29 November 2021) or bHaptics (https://www.bhaptics.com, last accessed on 29 November 2021). However, both haptic systems provide vibrotactile feedback solely on individual body parts rather than the whole body. Another solution for motion capture would be to employ special trackers, initially developed to bring any real-world object into virtual reality, such as HTC Vive trackers (https://www.vive.com/us/accessory/tracker3, last accessed on 29 November 2021). Although many different motion capture systems exist, the availability of haptic suits is only sparse. While substituting the Teslasuit would generally be possible, such an approach would nevertheless require two different systems (for motion capture and haptic feedback), possibly increasing the setup effort.

The basic approach for movement assessment is outlined as follows: To assess motor skills throughout activity execution, we first need to identify which exercise the user is currently performing (see Section 2.1). Afterward, we segment the sensor data and determine the start and end of every repetition. Segmentation is necessary to train probabilistic movement models to recognize exercise execution errors and finally provide haptic feedback (see Section 2.2).

### 2.1. Exercise Prediction

The main challenge before assessing movements is that we need to predict the exercise before the user actually performs it. Exercise prediction is necessary, as motion assessment and haptic feedback should be provided during the motion and as soon as an execution error occurs. Thus, in contrast to previous works that usually classify exercises after the repetition has been at least partially completed, we aim to determine an exercise before the repetition starts.

Towards this end, we classify exercises based on a starting pose of an exercise. Such an approach is indeed limited by the different starting poses for all exercises within the exercise pool and further requires a brief break between repetitions. Alternatively, we could define a training plan with predefined exercises that the user must adhere to. However, an approach with predefined exercises might be too restrictive for the users. Therefore, we instead allow users to decide which exercise they would like to do. For exercise classification, we train models using support vector machines (SVM), enabling us to differentiate between exercise starting poses and repetitions, as well as to identify movements that do not belong to an exercise at all. For classification, we use the following labels:

- *exercise_POSE*: indicating the starting pose of an exercise;
- *exercise_REP*: indicating the exercise repetition;
- *NEGATIVE_NONE*: indicating a default state, e.g., before and after the exercise.

#### Data Preprocessing and Feature Extraction

**Figure 3:**
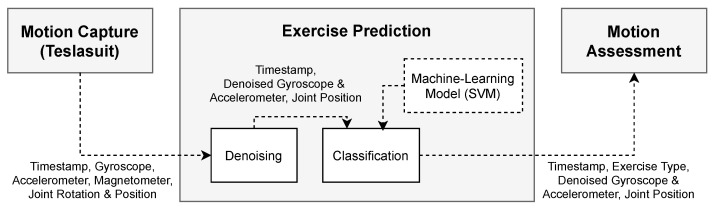
Flowchart for exercise prediction.

The Teslasuit SDK provides the raw sensor data of an accelerometer, a gyroscope, and a magnetometer. Additionally, the onboard control unit of the Teslasuit further uses an algorithm that reduces the sensor drift to infer the rotation of individual nodes, represented as a quaternion. Based on these data, the Teslasuit plugin (for Unity game engine) further estimates the human pose and reconstructs a virtual body that mimics the real body of the user wearing the suit. This avatar then enables us to obtain joint positions, represented as vectors. However, as magnetometer data depend on the user’s orientation, we employ only accelerometer (including the force of gravity) and gyroscope measurements for exercise prediction. Joint positions are used to train probabilistic movement models (see Section 2.2.2). A flowchart for exercise prediction is presented in Figure 3.

As the collected raw sensor data are generally subject to noise, we preprocess the data and smooth the measurements using a moving average filter with different window sizes, i.e., $T_{denoise}=\left\{1,2,\cdots ,10\right\}$. Denoising the signals is necessary to make the model more stable, reliable, and robust. At the first attempts at exercise prediction, we classify every single sample. To further improve the classifier performance, we could also calculate features over the sliding window, e.g., calculating mean, standard deviation, mean absolute deviation, interquartile range, and mean crossing rate, as already proposed by [19,24]. Nevertheless, as the sliding window approach causes delays (filtering over 10 samples at 50 Hz causes a delay of 200 ms), we would first need to determine from what point users notice the delay in haptic feedback.

### 2.2. Motion Assessment

#### 2.2.1. Segmentation

In order to assess the movement throughout the execution and provide direct feedback, we need to detect the start and end of each exercise repetition. Thereby, we use one three-axis gyroscope $g=(g_{1},g_{2},g_{3})$ to detect when users stand still and when they begin to move. We use a gyroscope attached to the right upper leg to detect squats or lunges, whereas we use gyroscope measurements of the right upper arm to detect push-ups.

**Figure 4:**
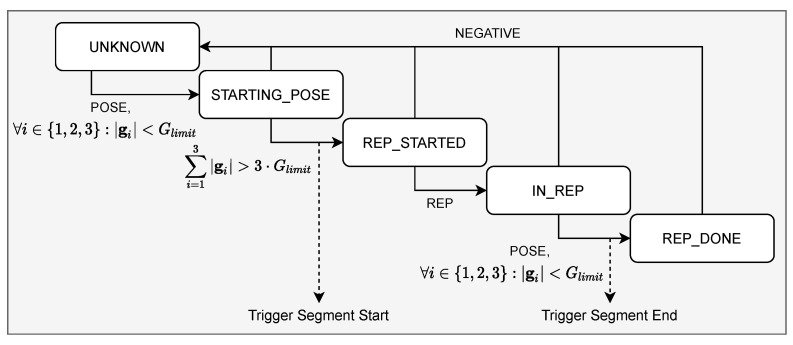
Algorithm for segmentation.

Additionally, we use results from the classification model, which labels each sample as *POSE*, *REP*, or *NEGATIVE*. As shown in Figure 4, the beginning of an exercise (i.e., *STARTING_POSE*) is detected as soon as the absolute values on each gyroscope axis are below a certain threshold: $\forall i\in \left\{1,2,3\right\}:|g_{i}|<G_{limit}$, whereby we test several values, i.e., $G_{limit}=\left\{1,2,\cdots ,15\right\}$, to determine an appropriate threshold. In other words, an exercise is identified when the user takes a stationary starting pose for a specific exercise.

Furthermore, we recognize when the user begins to move and starts the repetition, respectively, (i.e., *REP_STARTED*), when the sum of gyroscope measurements exceeds a certain threshold: $\sum_{i=1}^{3}\left|g_{i}\right|>3\;\cdot \;G_{limit}$. Similar to the recognition of the exercise beginning, we recognize the end of the repetition (i.e., *REP_DONE*) when the values of all three gyroscope axes are below a certain threshold.

#### 2.2.2. Training Probabilistic Movement Models

**Figure 5:**
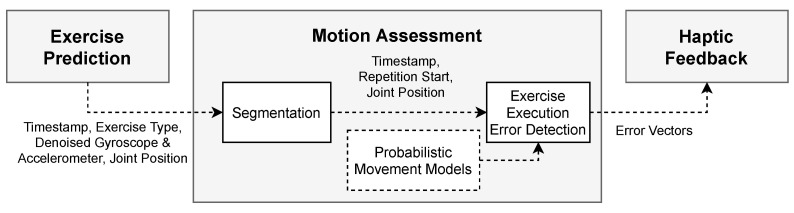
Flowchart for movement assessment.

After the exercise has been predicted and repetitions have been segmented, we train probabilistic movement models to detect exercise execution errors. Generally, probabilistic movement models represent a distribution over trajectories and previously showed promising results in robotics [25]. They are particularly suitable for human exercise assessment as they can learn a specific trajectory or movement based on expert demonstrations. Therefore, we exploit the advantage of imitation learning and build probabilistic movement models for each body part. Thereby, we use joint positions from the reconstructed avatar. As the Teslasuit plugin already estimates the human pose and applies the movements to a uniform avatar, we do not need to consider the user’s height and the lengths of the limbs. This enables us to compare motor skills between subjects, regardless of their height. A flowchart for movement assessment is visualized in Figure 5.

The idea of imitation learning enables detecting exercise execution errors in real-time without determining specific error classes in advance. In other words, we recognize execution errors by comparing the observed motor skills of users with a probabilistic model learned from expert demonstrations. Thus, in contrast to classification methods that only provide limited information on specific execution errors, whereas possible error classes need to be known a priori, we instead train probabilistic movement models for each IMU or joint. Probabilistic models also allow us to determine a poor exercise performance as soon as the joint position exceeds the standard deviation of the reference execution. In particular, such an approach enables us to detect the severity of the execution error, allowing us to provide stronger haptic feedback for more significant errors.

**Figure 6:**
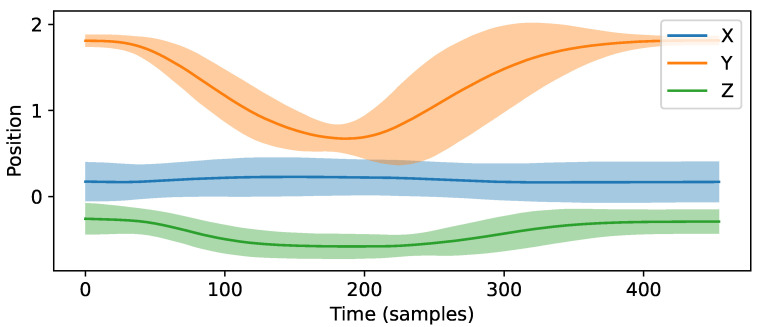
Example of a trajectory. The plot represents the mean position and two times the standard deviation (95% confidence interval).

Generally, probabilistic movement models require two matrices: (1) a matrix holding mean position values based on expert demonstrations $M\in R^{3\times n}$ and (2) a matrix holding standard deviation along the trajectory $S\in R^{3\times n}$, with *n* indicating the number of samples. The rows of the matrices hold the values for the *x*, *y*, and *z*-axis of the specified joint. To recognize execution errors, we need to compare the continuous sensor data stream, i.e., position vector $p\left[t\right]\in R^{3\times 1}$ at time step *t*, with the trained movement model, M and S, built from expert movements. The algorithm works as follows:

1. Calculate the absolute deviation $d\in R^{3\times 1}$ for each joint: $d=|M_{i}-p\left[t\right]|$, where $M_{i}$ represents the mapped mean position vector required for comparing the current sample. Because the duration of a single repetition is fixed and the repetition starting point is known, we can simply employ the Euclidean distance to determine the index *i*, based on the time elapsed since the start of the repetition.
2. Detect the movement error $e\in R^{3\times 1}$ for each joint:
  (1) $$e=\left\{\begin{array}{cc} 0, & \mathrm{if}\;d\leq k\;\cdot \;S_{i};\\ d-S_{i}, & \mathrm{else}. \end{array}\right.$$
  Herewith, *k* determines how much deviation from the experts’ demonstrations is allowed before detecting an error. We choose k=2 to set the level of confidence to approximately 95%. Thus, we identify an error when the deviation d is not within the confidence interval of the movement model. Figure 6 demonstrates one example of a probabilistic movement model.

#### 2.2.3. Providing Haptic Feedback

After the movement error for a specific joint and axis is determined (see Equation (1)), we need to calculate the location and intensity for the haptic feedback. Thereby, the challenge is to provide haptic feedback at the appropriate body location, which is not necessarily the position where the error occurs. For example, when the user’s elbow drifts outwards during a push-up, then the feedback should be triggered at the outside of the elbow to guide the user back into the intended position. The algorithm works as follows:

1. Determine the direction of an error $e_{direction}\in R^{3\times 1}$ for all axes (element-wise comparisons) as:
  (2) $$e_{direction}=\left\{\begin{array}{cc} e, & \mathrm{if}\;p\left[t\right]\geq M_{i};\\ e\;\cdot \;(-1), & \mathrm{if}\;p\left[t\right]<M_{i}. \end{array}\right.$$
2. Calculate the intensity of the error as: $e_{intensity}=e_{direction}/S_{i}\in R^{3\times 1}$.
3. Calculate the power for haptic feedback $h_{power}$. That is to say, we map the error vector $e_{intensity}$ to a value between zero and one, with zero indicating no haptic feedback and one indicating the highest intensity:
  (3) $$h_{power}=\left\{\begin{array}{cc} ∥e_{intensity}∥/f_{skill}, & \mathrm{if}\;∥e_{intensity}∥\leq f_{skill};\\ 1, & \mathrm{else}, \end{array}\right.$$
  Threshold $f_{skill}$ enables us to adapt the power of the haptic feedback to the user’s skills. For example, we can choose a larger threshold for patients to provide only slight sensations and a smaller threshold for athletes to increase the intensity.

## 3. Results

We first created a database (see Section 3.1) to evaluate the feasibility of the proposed approach, i.e., its ability to predict the exercises correctly (see Section 3.2), segmented the data (see Section 3.3), and assessed the motion to detect any execution errors without noticeable delay (see Section 3.4). The current system also provides haptic feedback based on the detected execution errors; however, the effectiveness of the haptic feedback was not evaluated within this study and should be done in future work. Therefore, it would be necessary to conduct an additional study, e.g., comparing the results of haptic feedback with a personal trainer.

### 3.1. Creating a Database

**Figure 7:**
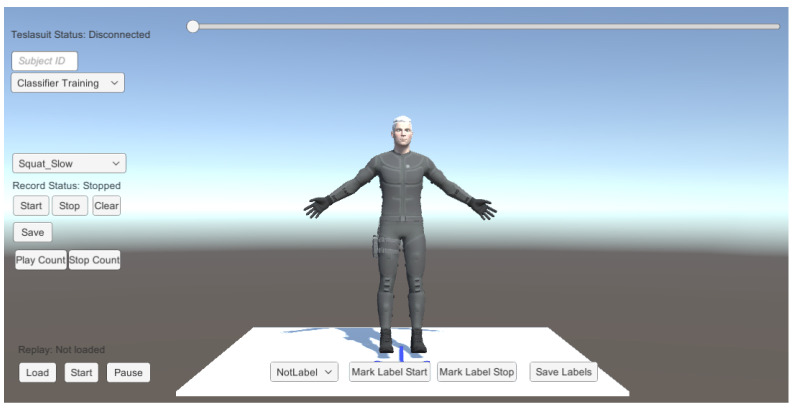
User interface, used for collecting sensor data.

We developed a Unity application (see Figure 7) to create a database for training and testing support vector machines and probabilistic movement models. Previous research suggests that a sample rate as low as 20 Hz is sufficient for human activity recognition [26,27]. The Teslasuit can stream data at frequencies of 28, 50, 100, and 200 Hz. Therefore, we set the sampling frequency to 50 Hz to minimize computational costs while staying well above the recommended minimum frequency. Hence, we collect the sensor data of all 10 nodes every 20 ms, i.e., saving the gyroscope, magnetometer, acceleration data, as well as the joint rotations and positions. We then verify the recorded sensor data by replaying the exercise repetitions and labeling them for the evaluation.

#### 3.1.1. Participants

We recruited 15 subjects (M=29.2, SD=4.13, between 22 and 38 years old, 3 female). Due to the size of the available Teslasuit, subjects had to be around 176–194 cm tall with a medium body size. The average subjects’ height was 180.4 cm (SD=6.65). Self-reports indicate an average weekly physical activity of 4.7 h (SD=3.17). At the beginning of the experiment, the participants read and signed the consent form. The study was approved by the local ethics committee of the Technical University of Darmstadt. Afterward, the subject first put on the Teslasuit and calibrated the motion capture sensors and haptic feedback.

#### 3.1.2. Procedure

All subjects were asked to perform three physical exercises described in the Functional Movement Screen [28]: deep squat, line lunge, and push-up. The Functional Movement Screen is particularly suitable for our purpose, as it describes the correct exercise execution and identifies dysfunctional movement patterns that could impair health and lead to injury. The subjects were instructed to repeat each exercise approximately four times at a constant repetition duration of 4 s, i.e., 2 s for concentric and 2 s for eccentric muscle action.

We further introduced breaks between each repetition: *long*, *short*, and *no* break. Thereby, the pause between two repetitions should last 2 s for the *long* and 1s for the *short* break. Moreover, to collect sensor data with *no* breaks between repetitions. Note that the movement repetition duration is equal for all three break conditions, i.e., 4 s. These “correct” repetitions were used for training the classifier (to predict the exercise) and probabilistic movement models (to assess the execution).

In addition to collecting the data of “correct” executions, subjects were also instructed to simulate poor performance and intentionally perform specific errors. These “incorrect” repetitions were then used to evaluate the feasibility of detecting execution errors. For *error type I*, subjects should lean their upper body too far forward when doing squats and lunges. For push-ups, subjects should first raise their shoulders and only bring the hips up later (arching the back), i.e., not keeping the body in a straight line. For *error type II*, subjects should cave their knees inward when doing squats. Furthermore, they should elevate their front heel and push their front knee as far forward as possible when doing lunges. For push-ups, subjects were instructed to flare their elbows. Lastly, for *error type III*, subjects should perform only partial repetitions, i.e., for every exercise, they should not lower the body deep enough. Between all repetitions, while simulating poor performance, subjects should take a break of 1 s. Table 1 further summarizes the different error types.

**Figure 8:**
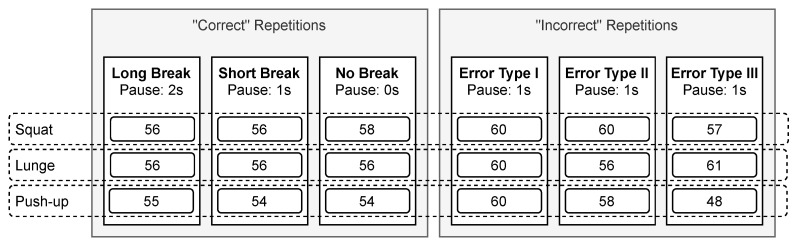
Training protocol with three sets of “correct” and “incorrect” repetitions, each. While the break conditions change between sets (2 s, 1 s, or 0 s), the repetition duration remains equal (4 s). The numbers in the boxes indicate the total number of repetitions collected from 15 subjects.

Because not all subjects could complete four clean repetitions successively, we thus had to discard some data. In total, our database consists of 163–170 “correct”’ and 166–177 “incorrect” repetitions per exercise at different break conditions (i.e., *long*, *short*, and *no* break). The sample number is comparable with related work on probabilistic movement models, using as little as 20 samples, whereas additional samples do not significantly improve the performance [25]. Figure 8 further shows the number of sets, number of repetitions, and break durations.

### 3.2. Exercise Prediction

To measure the error rate of the learned classification model, we apply leave-one-subject-out cross-validation. Generally, applying cross-validation counteracts overfitting, which occurs when a machine learning model fits the training data too tightly and performs poorly on unseen data. Therefore, we first train for each of the given *n* users a separate model using the data set of the n−1 remaining users. Afterward, we test the trained model using the data related to one user, previously not used for training. To report the success rate of a model, we specify the accuracy. Furthermore, as we have a multi-class problem, we calculate the macro-averaged $F_{1}$ scores over all subjects and additionally specify precision and recall.

**Figure 9:**
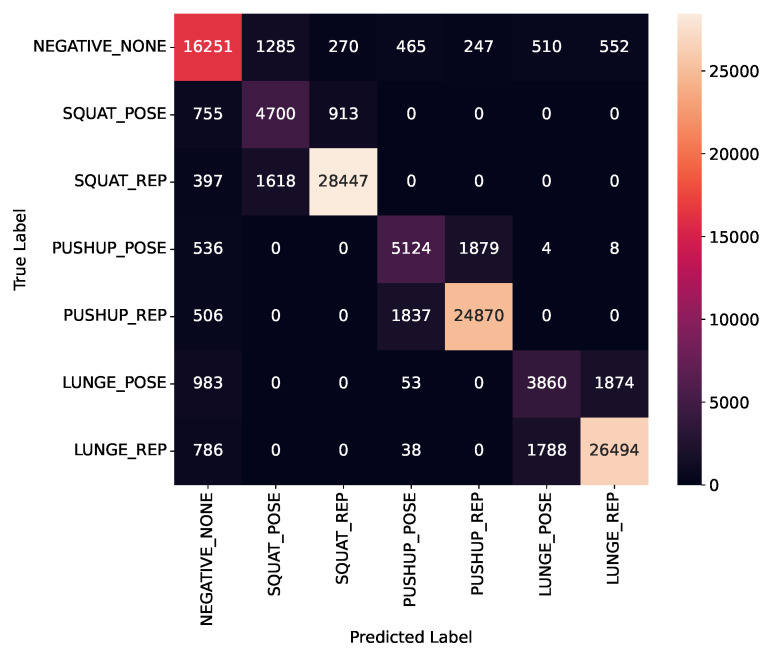
Confusion matrix for exercise classification.

Experiments on exercise classification using SVM showed that we obtain the best results when we do not filter sensor data at all, i.e., setting the window size to $T_{denoise}=1$. We achieve an accuracy of 86.38%; however, a lower $F_{1}$ score of 0.7920 (with precision equal to 0.7896 and recall equal to 0.7964). As shown in Figure 9, the main reason for the low $F_{1}$ score is that the model often confuses the starting pose with the repetition itself. With increasing window size up to $T_{denoise}=10$, the accuracy slightly decreases to 85.48% (with $F_{1}$ score decreasing to 0.7827, precision decreasing to 0.7814, and recall decreasing to 0.7867).

### 3.3. Segmentation

As it is challenging to accurately detect the start and end of the exercise repetition based on individual samples using a classifier (see Section 3.2), we additionally propose a segmentation algorithm. As described in Section 2.2.1, the segmentation algorithm depends on gyroscope measurements. The analysis revealed that for movements focusing on the leg muscle groups, such as squats or lunges, the gyroscope attached to the right thigh is the best choice. In addition, for movements that focus on arm muscle groups, such as push-ups, a gyroscope attached to the right upper arm is the best choice. Here, we again test different window sizes to smooth the data while keeping the $G_{limit}$ threshold equal to 2. We achieve the best results by setting the window size $T_{denoise}$ equal to 8. As data are sampled with 50 Hz, this means that the data are filtered roughly over the last 160 ms. Afterward, we also test different threshold values and achieve the best results with $G_{limit}$ equal to 8.

**Figure 10:**
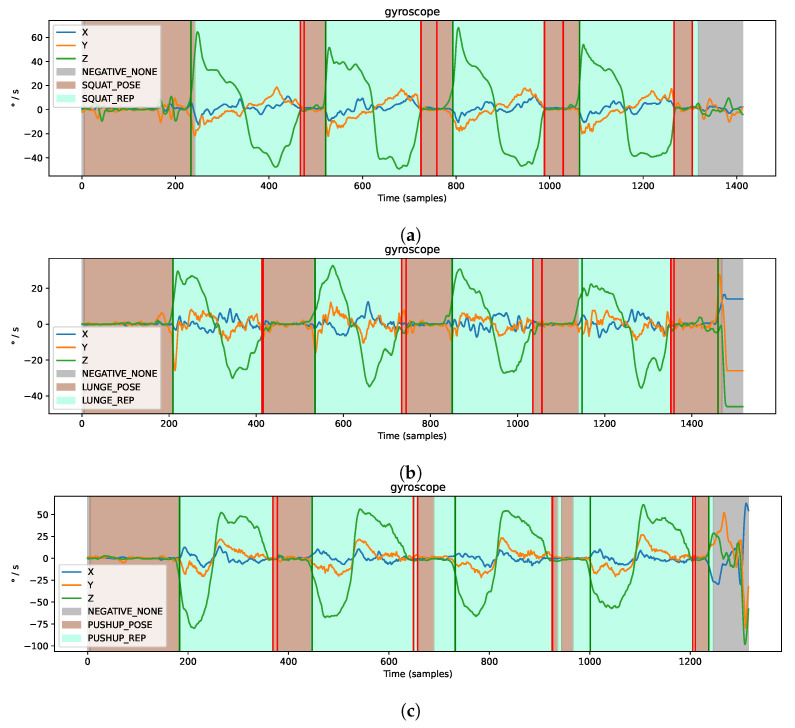
Examples for segmentation based on filtered gyroscope sensor data. Green lines mark the detected start and red lines mark the end of a repetition, (**a**) Right upper leg during the set of four squats (**b**) Right upper leg during the set of four lunges (**c**) Right upper arm during the set of four push-ups.

As shown in Table 2, the overall segmentation accuracy for the *long* break condition is 99.39% and for the *short* break condition, 96.98%. Thus, we can detect almost all exercises correctly if a pause of at least 1s is introduced between repetitions. However, without any breaks between exercises, we recognize fewer repetitions and the performance drops to 66.85%. These results confirm that the proposed algorithm on segmentation works best with a small break (of 1 or 2 s) between repetitions. Figure 10 further shows examples for segmentation (with long break conditions).

### 3.4. Execution Error Detection

During the sensor data collection and later during the evaluation, we observed that the recorded movements of some subjects were inadequate. Building probabilistic models using all “correct”’ repetitions would cause too much variety. Therefore, we build probabilistic movement models using only high-quality repetitions, selected by the expert. In total, we used 74 squats, 60 lunges, and 48 push-ups to build probabilistic movement models. Afterward, we use all 520 “incorrect” repetitions (see Figure 8) to evaluate the systems’ ability to detect execution errors.

**Figure 11:**
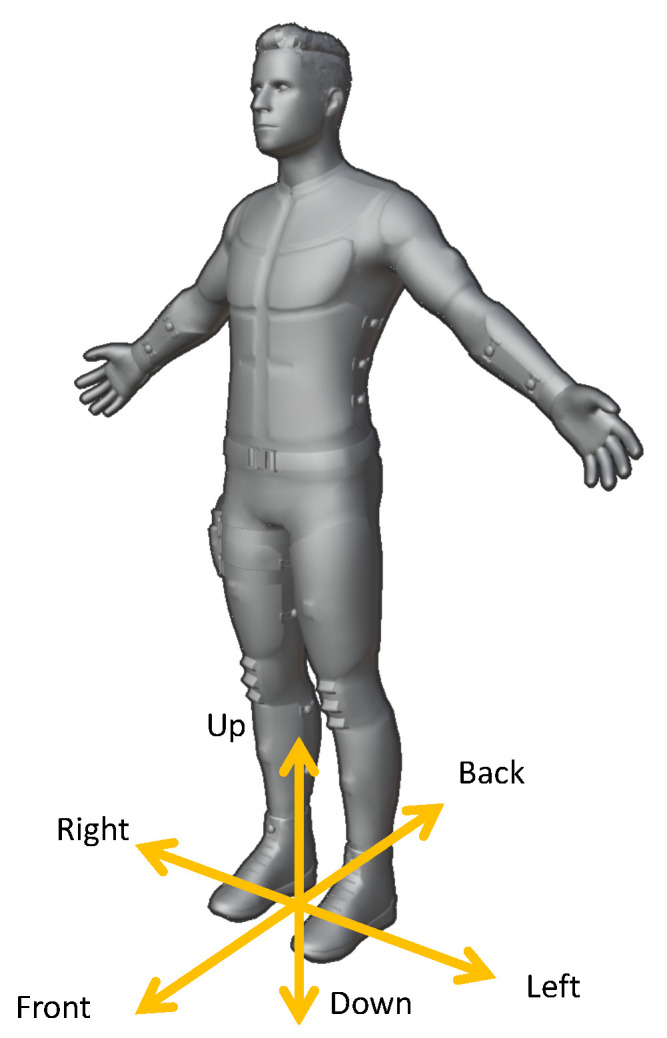
Directions used for binning of error vectors.

**Figure 12:**
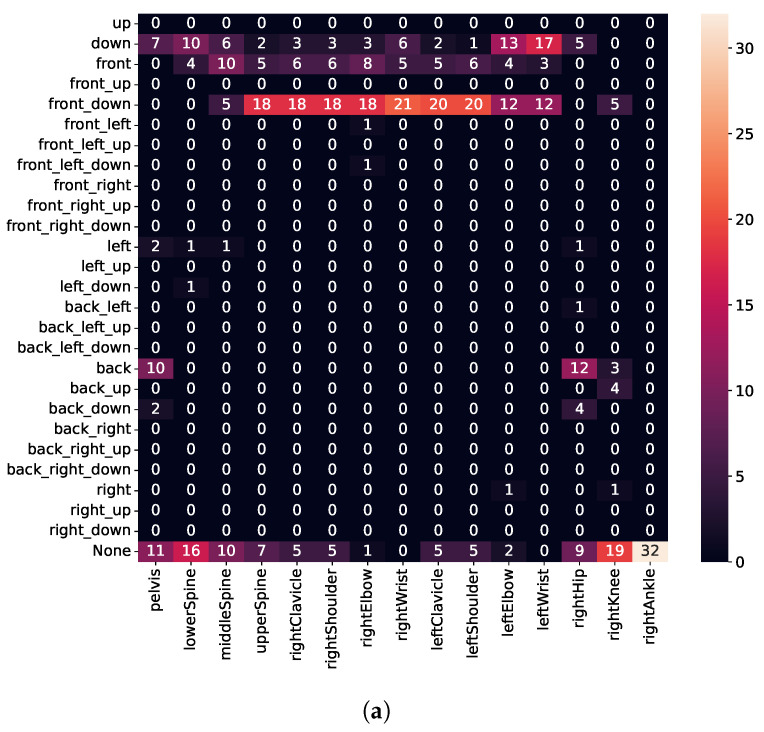

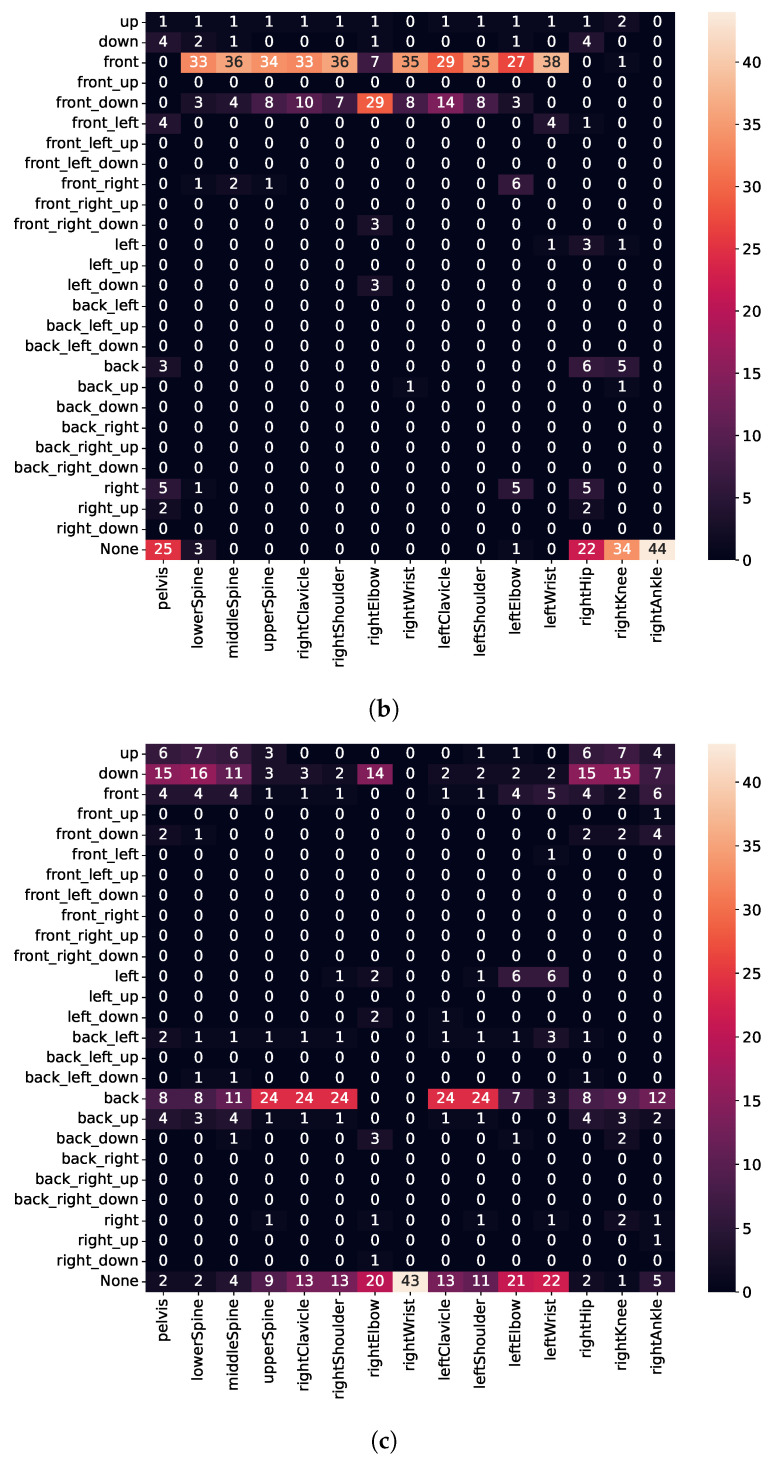
Detected execution errors for the *error type I*. (**a**) Squat; (**b**) Lunge; (**c**) Push-up.

**Figure 13:**
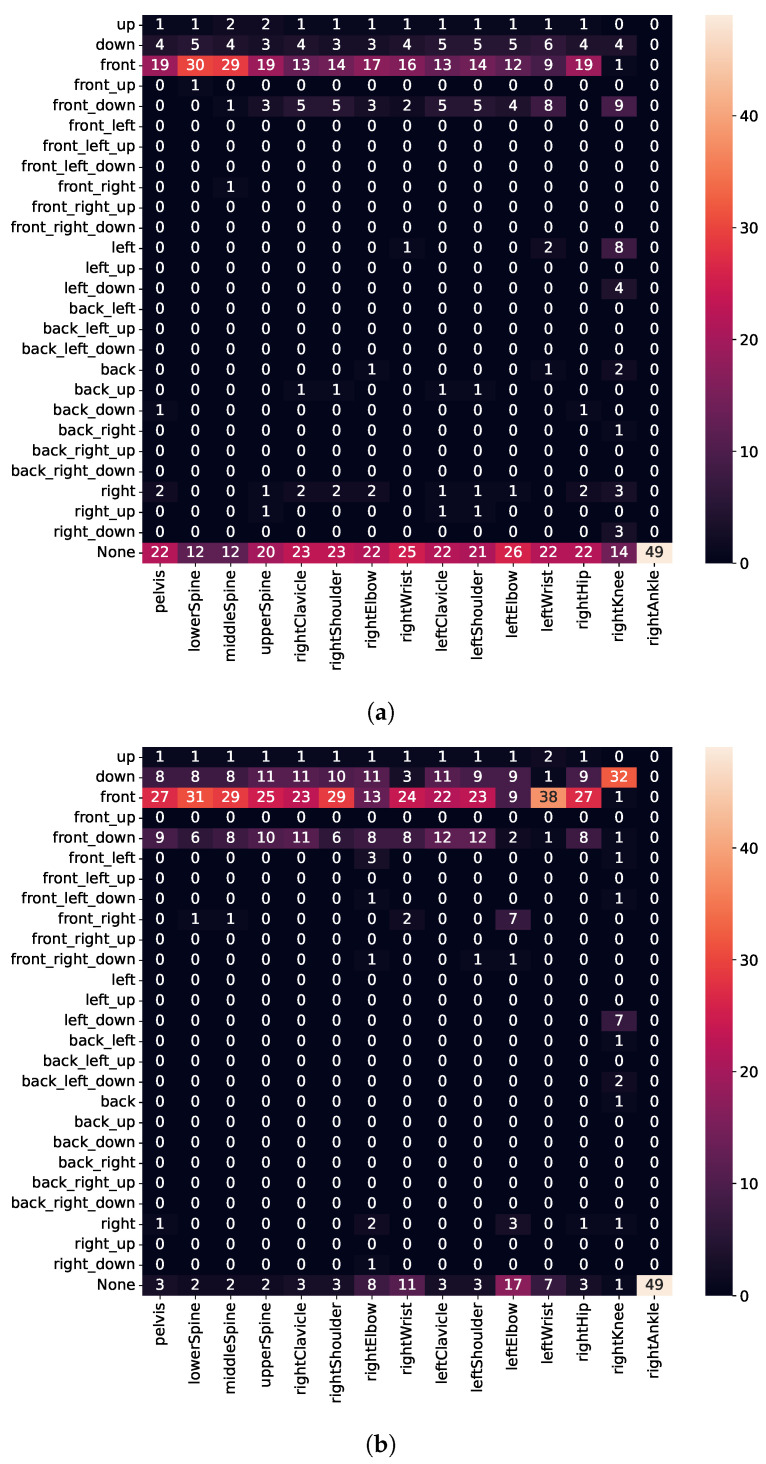

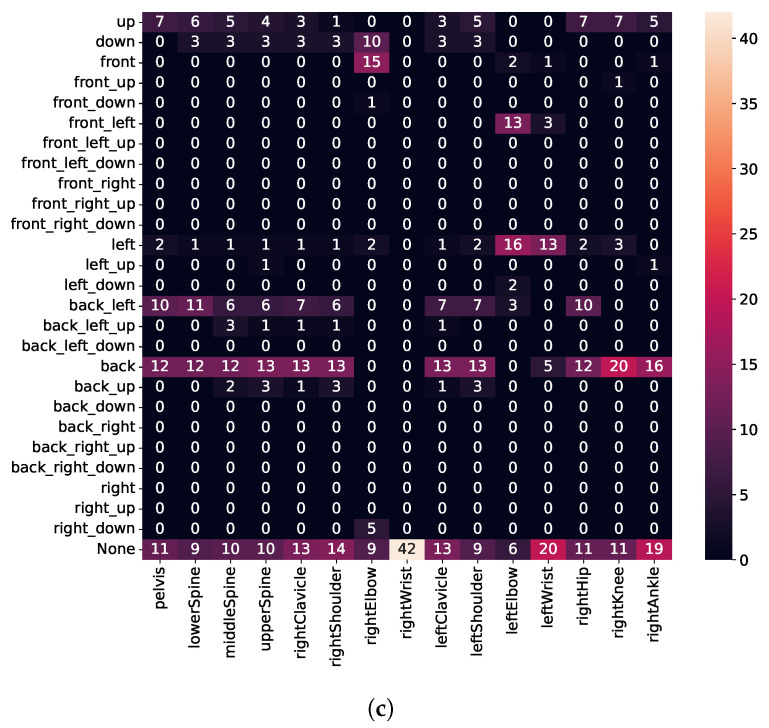
Detected execution errors for the *error type II*. (**a**) Squat; (**b**) Lunge; (**c**) Push-up.

**Figure 14:**
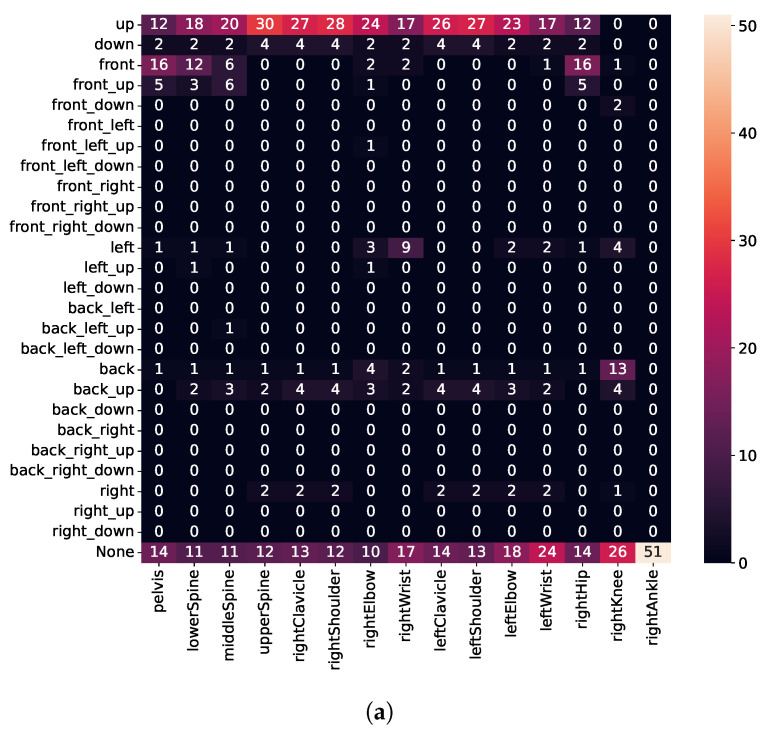

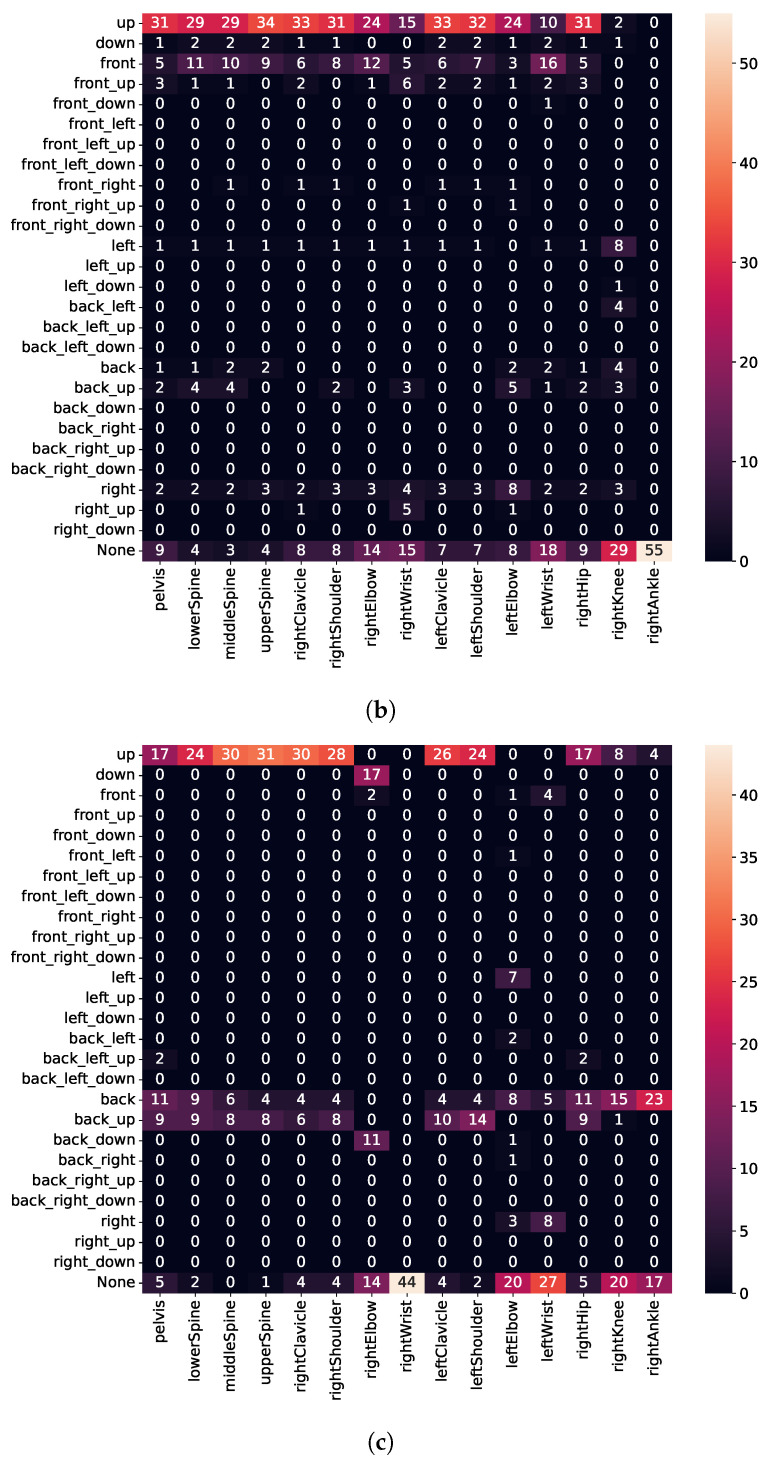
Detected execution errors for the *error type III*. (**a**) Squat; (**b**) Lunge; (**c**) Push-up.

To assess movements, we calculate the error vector (see Equation (2)) for every joint, represented in the fixed reference frame, as shown in Figure 11. Thus, the *top* and *down* directions are always aligned with the ceiling or floor, respectively, regardless of whether the user is standing or lying down. Unfortunately, due to a bug, we needed to discard sensor values for the left leg. Therefore, execution errors for the left leg are missing in Figure 12, Figure 13 and Figure 14. The Supplementary Video further demonstrates some of the recognized execution errors.

#### 3.4.1. Detecting Error Type I

Type I errors (see Table 1) include repetitions where subjects intentionally leaned their torso forward to perform squats and lunges, rather than keeping it upright. For the push-up, subjects were instructed to arch the back.

As detailed in Figure 12a, we detect when users lean their upper body too far forward for squats well (i.e., high intensity for *front-down* direction for upper-body joints), whereas the hips move backward. Although subjects were instructed to move only their upper body forward, we believe they unintentionally moved their hips back to maintain balance. Similarly, we also detect execution errors for lunges well (i.e., high intensity for *front* direction for upper-body joints), whereas no errors occur for lower-body joints (see Figure 12b). Furthermore, for push-ups (see Figure 12c), we detect when users keep the pelvis, lower spine, and hips on the floor (i.e., high intensity for *bottom* direction), whereas the upper spine, shoulder, and clavicle move backward (i.e., high intensity for *back* direction). Thus, we identify when users do not keep shoulders, hips, and feet in a straight line during the execution but instead arch their back.

#### 3.4.2. Detecting Error Type II

Type II errors (see Table 1) include repetitions, where subjects were instructed to cave their knees in for squats, push their front knee (always right) forward for lunges, and flare their elbows for push-ups. As shown in Figure 13a, we can only detect a few execution errors for the right knee (moving the knee in the *left* or *front-down* direction). We believe that the execution error for caving knees is not severe enough to be detected; however, it causes users to lean forward. Because no error is detected in many repetitions, this also indicates that we need to train further models with additional data from athletes or sports scientists.

For lunges (see Figure 13b), subjects shifted almost the whole body forward when instructed to push their front knee forward. Contrary to our expectations, we found that the right knee moves too far in the *bottom* direction instead of the *front* direction. We believe that because subjects lift their heel when pushing the knee forward, this causes the knee to move much more downward as forward. Finally, for push-ups (see Figure 13c), we detect that the left elbow drifts too far outwards (i.e., high intensity for *left* and *front-left* direction); whereas, the right elbow drifts in the *forward* or *down* direction. Although subjects were instructed to keep their back straight, we also observe that some subjects nonetheless could not meet this requirement. Nevertheless, the error execution for *error type II*, i.e., not keeping the back straight, seems to be less severe compared to *error type I* (comparing Figure 12c and Figure 13c).

#### 3.4.3. Detecting Error Type III

For the type III errors, subjects were instructed to perform partial repetitions and not to lower the body deep enough. As depicted in Figure 14, we detect that most joints are kept too far above for all three exercises (i.e., high intensity for *top* direction). Moreover, for squats and lunges, we detect no error for ankle and knee. This is in line with the expectation, as knee or ankle height does not change, regardless of how deep the user performs a squat or a lunge. Similarly, also for push-ups, we detect that the upper spine, shoulders, and clavicle are kept too far above, while no error for wrists is detected.

## 4. Discussion

In contrast to previous works on exercise assessment, often analyzing and classifying an exercise only after the repetition has been at least partially completed, we intend to provide feedback already during the motion or as soon as an error occurs. The results show that we can correctly predict up to 86.38% of the exercises (see Section 3.2). Furthermore, depending on the exercise, we segment 96.98% to 99.39% of the exercises correctly (see Section 3.3) when users take a short break between repetitions (1–2 s).

Moreover, by analyzing movements on individual joints, we overcome limitations of related works that often only assess if a specific exercise was performed well or poorly. Instead, we intend to recognize arbitrary execution errors (without specifying them a priori) and provide haptic feedback at appropriate body positions to assist users in correcting their motor skills. Our results on motion assessment show that the system is able to detect many execution errors, such as leaning the upper body forward while performing squats and lunges or not keeping the back straight while doing push-ups. Towards this end, we trained probabilistic movement models only on positive repetitions (samples labeled as “correct”’) and tested them on negative repetitions (error types as specified in Table 1). These results indicate that the detected errors occur due to the difference between “correct” and “incorrect” movement rather than variations in “correct” executions.

However, more minor errors could be only partially recognized, such as caving the knees during squats or flaring the elbows during push-ups. To also detect these minor execution errors, we need to train more accurate models. For example, we could integrate personal models, as they have previously shown to perform better than generic or hybrid models [29,30]. On the one hand, generic models are expected to perform well for any user, as they are trained and tested on different users. On the other hand, personal models use only one user’s data and are thus tailored to one user. The models trained in this paper use the data of multiple users, causing large deviations due to differences in muscular strength or flexibility in users. To overcome this limitation, we could also employ personal models to learn user-specific differences more effectively, as already pinpointed by Lockhart and Weiss [30]. However, personal models require collecting sensor data from each user and re-training the probabilistic movement models. Therefore, we could initially build models from experts and gradually collect the movements of individual users during their training. Such an approach would also enable us to improve or correct the models as users progress.

Overall, the evaluation results show that we are capable of detecting execution errors in real time. A possible application scenario for the proposed method includes, e.g., individual training at home, whereby users could borrow a suit at a rehabilitation center. Such an application scenario would be especially suitable at the present time, during the COVID-19 pandemic, e.g., to motivate patients to repeat specific exercises regularly and independently in a home environment, even during a lockdown. Thereby, the exercises’ repetition and execution could also be monitored through full-body motion recognition by the therapist.

### Limitations

We aimed to assess full-body movements in real-time; however, this paper presents several limitations. Firstly, the sample’s size is relatively small as we needed to discard many repetitions due to poor performance. As none of the subjects were professional athletes, many had difficulty performing all exercises as instructed by the supervisor, primarily due to low mobility or insufficient strength. For example, during squats, we could observe that subjects often could not keep their arms outstretched, whereas, during lunges, subjects often had trouble keeping their balance. Therefore, in future work, we intend to additionally collect data from athletes or sports scientists.

Secondly, the probabilistic movement models are limited by the repetition duration. In our experiments, we collected sensor data while subjects were performing exercises at a given speed (i.e., 2 s for concentric and eccentric muscle action). Such an approach, enforcing a specific repetition duration, might be beneficial and desired, e.g., for muscle activation [31] or during resistance training [32], although there is some contradictory evidence [33,34]. However, to recognize movements exercises at any tempo, we would need to adapt the trained models. To overcome this limitation of fixed repetition duration, we could employ dynamic time warping, as they already showed promising results in gesture recognition [35].

Thirdly, in this paper, we did not evaluate the effectiveness of haptic feedback. In future work, we intend to evaluate the implemented haptic feedback by a personal trainer, who can provide further evidence. Despite these limitations, we could nevertheless demonstrate the feasibility of the system, using a full-body motion capture suit with haptic feedback to assess movements in real time and recognize severe exercise execution errors.

## 5. Conclusions

In this paper, we propose an approach for full-body movement assessment in real time. We overcome the limitations of many existing health or fitness applications, which specify execution errors in advance, focus on movements of a specific body part, or provide feedback first after the repetition. We instead detect execution errors directly and provide haptic feedback at relevant body locations throughout the execution. The results of the experiments show that we predict exercises with an average accuracy of 86.38%. Furthermore, we segment 99.39% or 96.98% of repetitions correctly if a break of 2 s or 1 s is introduced between them, respectively. Moreover, evaluation on the movement assessment shows that we are able to detect severe execution errors by building probabilistic movement models across multiple joints. Although we could demonstrate the feasibility of motion capture technology and haptic feedback for movement assessment, current work still has potential for improvements with respect to the recognition performance. In particular, to detect minor execution errors more accurately, we would need to train further models using data from athletes or sports scientists.

## Figures and Tables

**Table 1:** Three types of errors.

Exercise	*Error Type I*	*Error Type II*	*Error Type III*
Deep squat	Torso leaned forward	Inward knees	Not deep enough
Line lunge	Torso leaned forward	Front knee pushed forward	Not deep enough
Push-up	Arched back	Flared elbows	Not deep enough

**Table 2:** Segmentation accuracy per exercise and break condition.

Exercise	*Long Break* (2 s)	*Short Break* (1 s)	*No Break* (0 s)
Squat	100%	96.43%	56.90%
Lunge	100%	98.21%	71.43%
Push-up	98.18%	96.30%	72.22%
Average	99.39%	96.98%	66.85%

## Data Availability

Source code and (anonymized) datasets are available online at: https://github.com/serious-games-darmstadt/MovementAssessmentWithTeslasuit (accessed on 29 November 2021).

## Supplementary Materials

The following are available online at https://www.mdpi.com/article/10.3390/s21248389/s1, The videos presented in the paper can be found from the link.

## Abbreviations

The following abbreviations are used in this manuscript:

IMU	Inertial Measurements Unit
SVM	Support Vector Machines
M	Medium
SD	Standard Deviation
SDK	Software Development Kit
